# Persistent activation of central amygdala CRF neurons helps drive the immediate fear extinction deficit

**DOI:** 10.1038/s41467-020-14393-y

**Published:** 2020-01-22

**Authors:** Yong S. Jo, Vijay Mohan K. Namboodiri, Garret D. Stuber, Larry S. Zweifel

**Affiliations:** 1 Department of Psychiatry and Behavioral Sciences, University of Washington, 1959 NE Pacific Street, Seattle, WA 98195 USA; 2 Department of Psychology, Korea University, 145 Anam-ro, Seongbuk-gu, Seoul, 02841 Republic of Korea; 3 Department of Anesthesiology, University of Washington, 1959 NE Pacific Street, Seattle, WA 98195 USA; 4 Department of Pharmacology, University of Washington, 1959 NE Pacific Street, Seattle, WA 98195 USA

**Keywords:** Neuroscience, Fear conditioning, Neural circuits, Stress and resilience

## Abstract

Fear extinction is an active learning process whereby previously established conditioned responses to a conditioned stimulus are suppressed. Paradoxically, when extinction training is performed immediately following fear acquisition, the extinction memory is weakened. Here, we demonstrate that corticotrophin-releasing factor (CRF)-expressing neurons in the central amygdala (CeA) antagonize the extinction memory following immediate extinction training. CeA-CRF neurons transition from responding to the unconditioned stimulus to the conditioned stimulus during the acquisition of a fear memory that persists during immediate extinction training, but diminishes during delayed extinction training. Inhibition of CeA-CRF neurons during immediate extinction training is sufficient to promote enhanced extinction memories, and activation of these neurons following delay extinction training is sufficient to reinstate a previously extinguished fear memory. These results demonstrate CeA-CRF neurons are an important substrate for the persistence of fear and have broad implications for the neural basis of persistent negative affective behavioral states.

## Abstract (web summary)

Learned conditioned fear associations can be weakened (extinction learning), but extinction is less effective if performed too soon after the original fear conditioning. Here, the authors show that persistent activation of CRF-expressing neurons in the central amygdala is involved in the early fear extinction deficit.

## Introduction

During Pavlovian fear conditioning, pairing a sensory stimulus (e.g., auditory, olfactory, or visual) with an unconditioned stimulus (US) results in a conditioned response (CR) following presentation of the associated conditioned stimulus (CS) that is typically measured as freezing or immobility in rodents^1^. Extinction of CS-evoked CRs can be achieved by repeated presentations of a CS in the absence of the US. This extinction is proposed to result from the formation of a new inhibitory memory that competes with a weakened fear memory^2^. In contrast to the predicted efficiency of extinguishing fear memories immediately following the CS-US association, extinction training that occurs proximal to conditioning is less effective than extinction training that follows a delay^3–8^.

It has been proposed that this immediate fear extinction deficit is the result of an active fear state and associated stress-arousal systems linked to the recency of fear that antagonizes the extinction process^6^. Evidence points to the involvement of both noradrenergic and corticotrophin-releasing factor (CRF) systems in the modulation of neural circuits underlying the immediate extinction deficit^4,5,8^, but how neurons involved in the early acquisition of the fear memory contribute to the immediate extinction deficit is not resolved.

The central amygdala (CeA) has emerged as a critical site in the early formation of fear memories^9–13^. CeA neurons that produce the stress-associated neuropeptide CRF are an important regulator of conditioned fear responses and the scalability of fear^14,15^. CeA–CRF neurons also potently modulate noradrenergic neurons of the locus coeruleus (LC)^16^ and LC noradrenergic neurons potently modulate the BLA^17^, a brain region where noradrenergic signaling is linked to the immediate extinction deficit^5^. These findings suggest that CeA–CRF neurons may represent a key component of the neural circuitry associated with immediate extinction deficits associated with the recency of fear.

In support of our hypothesis that CeA–CRF neurons contribute to the immediate fear extinction deficit, silencing these neurons during immediate fear extinction significantly attenuates the extinction deficit. Imaging calcium dynamics in CeA–CRF neurons during threat conditioning, extinction, and extinction recall revealed that a large number of these neurons transition from US- to CS-responsiveness during conditioning. This CS-responsiveness persists during immediate fear extinction training and during extinction recall. In contrast, CeA–CRF neurons display diminished responsiveness to the CS during delayed fear extinction training and during recall. Finally, activation of these neurons during delay extinction is sufficient to induce an extinction deficit and to reinstate a previously extinguished conditioned threat response.

## Results

### CRF neurons contribute to the immediate extinction deficit

**Fig. 1:**
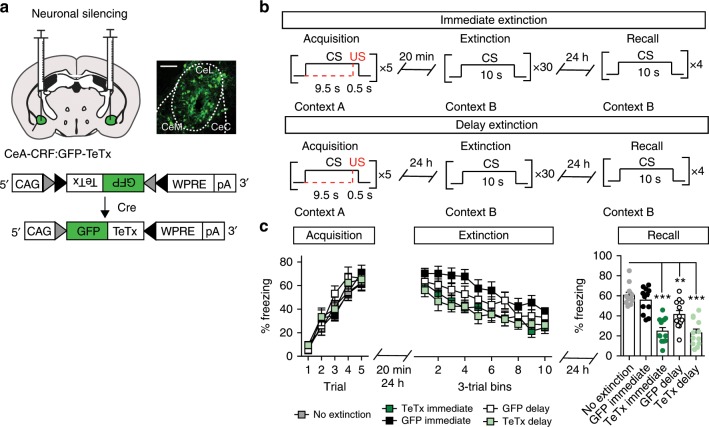
CeA–CRF neurons antagonize immediate fear extinction. **a** Schematic of bilateral injection of AAV1-FLEX-GFP-TeTx into the CeA and representative histological verification of expression. Scale bar: 250 μm. CAG cytomegalovirus (C) chicken beta actin exon 1/intron 1 (A) rabbit beta-globin splice acceptor (G) promoter/enhancer, WPRE woodchuck hepatitis virus posttranscriptional regulatory element, pA: bovine growth hormone polyadenylation sequence. **b** Experimental paradigm: Mice (*n* = 12/group) were conditioned with five presentations of an auditory CS that co-terminates with a 0.5 mA foot shock (0.5 s) in context A. During extinction, 30 unpaired CS presentations are delivered 20 min following conditioning in context B (immediate, top) or 24 h after conditioning (delay, bottom). **c** GFP-TeTx expression in CeA–CRF neurons does not alter acquisition (left) or extinction training (middle), but significantly enhances fear extinction recall (right) relative to the no ext. group (One-way ANOVA followed by Bonferroni multiple comparisons ***P < 0.001, **P < 0.01). This effect was not observed in GFP-expressing mice. Data are presented as mean ± Standard Error of the Mean (S.E.M.).

To test whether CeA–CRF neurons contribute to the immediate extinction deficit, we selectively silenced these cells by blocking synaptic transmission through expression of tetanus toxin light chain (TeTx)^13,18^. Adeno-associated virus (AAV1) containing a conditional expression cassette for the GFP-TeTx fusion protein (AAV1-FLEX-GFP-TeTx)^13^ was bilaterally injected into the CeA of *Crh*^*IRES-Cre*^ (CRF-Cre) mice^19^ (Fig. 1a). CeA–CRF:GFP-TeTx and CeA–CRF:GFP controls were fear conditioned using a US intensity (0.5 mA. 0.5 sec) previously shown to elicit CRF-independent acquisition of a fear-associated CR^15^. CS-evoked freezing was measured in five independent groups (12 mice/group) following acquisition training and either immediate or delay extinction training (Fig. 1b). These groups consisted of mice that underwent no extinction, immediate extinction (CeA-CFR:GFP, GFP immediate; CeA–CRF:GFP-TeTx, TeTx immediate), or delay extinction (CeA-CFR:GFP, GFP delay; CeA–CRF:GFP-TeTx, TeTx delay). All groups showed equivalent acquisition of CS-evoked freezing (Fig. 1c, two-way repeated measures ANOVA, group × time interaction, F_*(16,220)*_ = 0.73, *P* = 0.76). We also did not observe a significant interaction between group and extinction training, averaged as three-trial bins (Fig. 1c, two-way repeated measures ANOVA, group × time interaction, F_*(27,396)*_ = 0.60, *P* = 0.95) consistent with previous data demonstrating a lack of interaction between groups with immediate and delay fear extinction training^7^. Twenty-four hours following extinction training, mice were tested for extinction recall. GFP delay, TeTx immediate, and TeTx delay groups all displayed significantly less freezing than the no ext. control group (one-way ANOVA, F_*(4,55)*_ = 22.34, *P* < 0.0001).

**Fig. 2:**
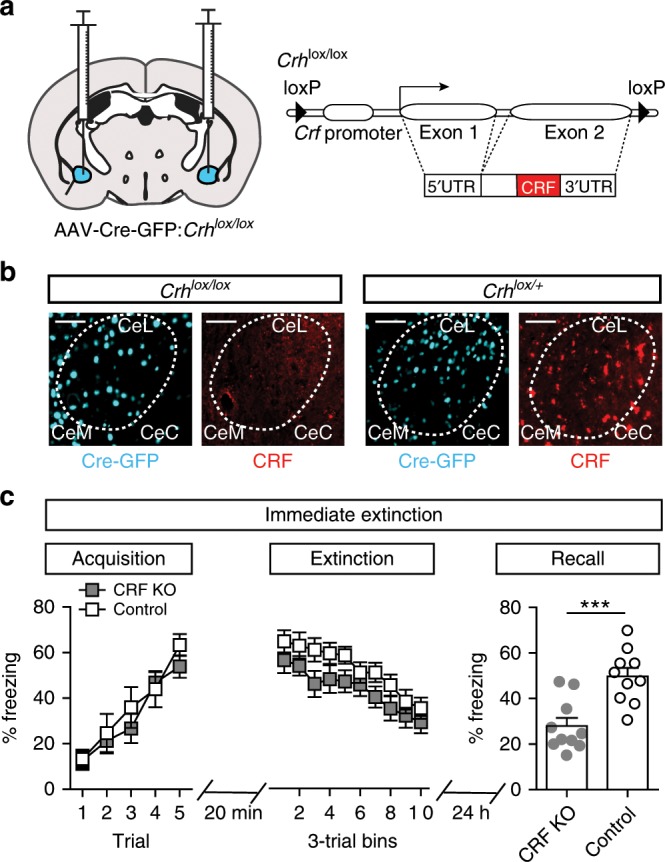
Inactivation of Crh in the CeA attenuates the immediate extinction deficit. **a** Schematic of AAV1-Cre-GFP injection into the CeA of *Crh*^*lox/lox*^ mice and corresponding loxP-flanked *Crh* allele. **b** Histological verification of Cre-GFP expression in the CeA and loss of CRF peptide in *Crh*^*lox/lox*^ injected mice. Scale bar: 100 μm. **c** Acquisition (left) and extinction (middle) are not altered by *Crh* inactivation in the CeA, but fear extinction recall (right) is significantly enhanced relative to *Crh*^*lox/+*^ mice injected with AAV1-Cre-GFP (Unpaired Student’s *t*-test, ***P < 0.001; *n* = 10/group). Data are presented as mean ± S.E.M.

To determine whether CRF produced by CeA–CRF neurons is involved in the immediate extinction deficit, we conditionally inactivated the CRF-encoding gene (*Crh*^*lox/lox*^)^15^ by injection of AAV-Cre-GFP into the CeA of *Crh*^*lox/lox*^ or *Crh*^*lox/+*^ control mice (Fig. 2a, b). Mice underwent acquisition training as above (10 mice/group), followed by immediate extinction training and extinction recall testing (Fig. 2c). We did not observe significant effects of CRF knockout (CRF KO) on the acquisition of CS-evoked freezing (Fig. 2c, two-way repeated measures ANOVA, group × time interaction, F_*(4,72)*_ = 0.93, *P* = 0.79) or CS-evoked freezing during immediate extinction training (Fig. 2c, two-way repeated measures ANOVA, group × time interaction, F_*(9,162)*_ = 0.26, *P* = 0.98). However, we did observe a significant reduction in freezing during extinction recall in CRF KO mice compared to controls (Fig. 2c, unpaired Student’s *t*-test, t_*(18)*_ = 4.22, *P* = 0.005).

**Fig. 3:**
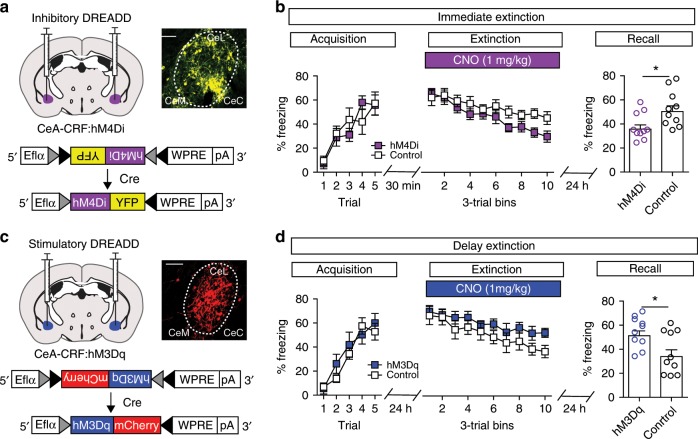
DREADD manipulation of CeA–CRF neurons during fear extinction. **a** Schematic of bilateral injection of AAV1-FLEX-hM4Di-YFP into the CeA of CRF-Cre mice (inset: immunohistochemistry for hM4Di-YFP expression pseudo-colored yellow, scale bar: 250 μm). Ef1α elongation factor 1 alpha promoter. Control mice were injected with AAV1-FLEX-YFP. **b** CNO (1 mg/kg) was injected (*n* = 10/group) immediately following acquisition training (5 CS-US pairings) followed 30 min later by extinction training 30 CS-only presentations. The next day (24 h), 4 CS-alone trials were given to test the recall of extinction memory. Acquisition (left) and extinction (middle) are not altered by CeA–CRF neuronal inhibition during extinction training, but fear extinction recall (right) is significantly enhanced (Unpaired Student’s *t*-test, *P < 0.05). **c** Bilateral injection of AAV1-FLEX-hM3Dq-YFP into the CeA of CRF-Cre mice (inset: immunohistochemistry for hM3Dq-mCherry expression pseudo-colored red, scale bar: 250 μm). Control mice were injected with AAV1-FLEX-mCherry. **d** CNO (1 mg/kg) was injected (*n* = 10/group) 30 min prior to delay extinction training (24 h after acquisition). Acquisition (left) and extinction (middle) are not altered by enhancing CeA–CRF neuronal activity during delay extinction training, but fear extinction recall (right) is significantly impaired (Unpaired Student’s *t*-test, *P < 0.05). Data are presented as mean ± S.E.M.

We previously demonstrated that CRF-producing neurons of the CeA regulate the acquisition of fear memory in response to weak, but not strong US conditioning^15^. It is possible that TeTx-silenced CeA–CRF and CeA–CRF KO mice have weakened fear acquisition memories that are not manifest in the acquisition of the CS-evoked freezing or the extinction training, but this weakened memory allows for an enhanced consolidation of the competing immediate extinction memory and improved extinction recall. To test this possibility we selectively inhibited CeA–CRF neurons only during the immediate extinction training through expression of the inhibitory DREADD^20^ receptor hM4Di (Fig. 3a). CRF-Cre mice were injected bilaterally with AAV1-FLEX-hM4Di-YFP or AAV1-FLEX-YFP (control). Following acquisition training (10 mice/group), mice were injected with clozapine-N-oxide (CNO, 1 mg/kg, i.p.) and underwent immediate extinction training 30 min following injection (Fig. 3b). There was no significant difference between groups during acquisition training (Fig. 3b, two-way repeated measures ANOVA, group × time interaction, F_*(4,72)*_ = 1.34, *P* = 0.26). CNO administration did not significantly alter CS-evoked freezing during immediate extinction training (Fig. 3b, two-way repeated measures ANOVA, group × time interaction, F_*(9,162)*_ = 0.97, *P* = 0.47). CeA–CRF neuron silencing with CNO during immediate extinction training resulted in a significant improvement in fear extinction recall (Fig. 3b, unpaired Student’s *t*-test, t_*(18)*_ = 2.49, *P* = 0.022).

If the activity of CeA–CRF neurons contributes to the immediate extinction deficit, then enhancing the activity these neurons during delay extinction training should impair extinction memory recall. To test this, we injected CRF-Cre mice with an AAV for conditional expression of the stimulatory DREADD receptor hM3Dq^20^ (AAV1-FLEX-hM3Dq-mCherry, Fig. 3c) or AAV1-FLEX-mCherry (control) and administered CNO during delay extinction training 24 h after acquisition training (Fig. 3d). Acquisition of CS-evoked freezing was not different between drug-naïve groups (Fig. 3d, two-way repeated measures ANOVA, group × time interaction, F_*(4,72)*_ = 0.87, *P* = 0.49, *n* = 10 mice/group). CNO administration did not significantly alter CS-evoked freezing during delay extinction training (Fig. 3d, two-way repeated measures ANOVA, group × time interaction, F_*(9,162)*_ = 0.45, *P* = 0.91), but did result in a significant impairment of the extinction memory in hM3Dq expressing mice tested 24 h later in a drug-free state (Fig. 3d, unpaired Student’s *t*-test, t_*(18)*_ = 2.25, *P* = 0.021).

### Persistence of CS activity during immediate extinction

**Fig. 4:**
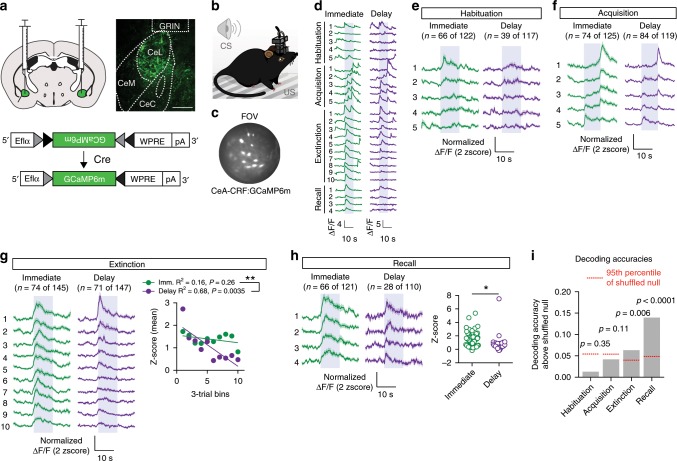
Imaging CeA–CRF neurons during fear conditioning and extinction. **a** AAV1-FLEX-GCaMP6m injection into the CeA and representative immunohistochemical verification of GCaMP6m expression and GRIN lens placement in the CeA. Scale: 250 μm. CeM central amygdala medial, CeL lateral, and CeC capsule. **b** Cartoon illustrating mouse with miniscope implantation and conditioning chamber. **c** Fluorescence field-of-view (FOV) from miniscope of GCaMP6m-expressing CeA–CRF neurons. **d** Representative individual cell responses during each of the four sessions, habituation, acquisition, extinction, or recall, blue bar denotes CS presentation. **e** Average normalized calcium (±S.E.M.) signals of CeA–CRF neurons that responded to the initial CS presentation during habituation (*n* = 24, 38, and 4 cells from each mouse immediate extinction and *n* = 13, 21, and 5 cells from each mouse delay extinction). **f** Average normalized calcium signals (±S.E.M.) of CeA–CRF neurons that were activated during acquisition (*n* = 37, 33, and 4 cells from each mouse immediate extinction and *n* = 43, 35 and 6 cells from each mouse delay extinction). **g** Average normalized calcium signals (±S.E.M.) of CeA–CRF neurons that responded to the CS presentation during extinction (*n* = 26, 36, and 12 cells from each mouse immediate extinction and *n* = 25, 35 and 11 cells from each mouse delay extinction). Linear regression analysis of the mean calcium signal (averaged across 5 s period following CS onset) and extinction trial was significant for the delay extinction group (*P* = 0.0035), but not the immediate extinction group (*P* = 0.26). There was a significant difference between the slopes (**P = 0.01). **h** Average normalized calcium signals (±S.E.M.) of CeA–CRF neurons that responded to the CS presentation during recall (*n* = 12, 35, and 19 cells from each mouse immediate extinction and *n* = 15, 7 and 6 cells from each mouse delay extinction). The average CS-evoked response (averaged across 5 s period following CS onset, ±S.E.M.) across the four trials is significantly different between the groups (Unpaired Student’s *t*-test, *P < 0.05). **i** CS-responsive cells during extinction training and recall were accurate at decoding behavioral groups relative to the shuffled null distribution. CS-responsive cells during habituation and acquisition were not significantly accurate decoders. P-values shown are one-tailed since the alternative hypothesis is accuracy, which is higher than chance.

Neurons of the CeA rapidly acquire responses to a CS during fear conditioning and reduce responsiveness following extinction^21^. Somatostatin neurons of the CeA (CeA-SOM) have also been shown to display increased calcium responses to a CS following conditioning^22^ and PKCδ-expressing neurons of the CeA show robust calcium responses to the US during conditioning, and a proportion of these neurons show CS responses during fear recall^23^. CeA–CRF neurons show CS-evoked increases in calcium^15^ and time-locked increases in action potential firing to a CS following conditioning^14^. To establish the response profile of CeA–CRF neurons during habituation, conditioned fear acquisition, extinction, and extinction recall we tracked calcium signals in these neurons during all four phases of behavioral testing. CRF-Cre mice were injected into the CeA with a Cre-dependent expression cassette for the genetically encoded calcium indictor GCaMP6m^24^ (AAV1-FLEX-GCaMP6m, Fig. 4a). Two weeks following viral injection a GRIN lens (0.6 mm diameter) was implanted above the structure (Fig. 4a). Ten days following GRIN lens implantation mice were calcium signals were acquired (Fig. 4b, c) using a miniature microscope^25^ as described^26^ during habituation, acquisition, extinction, and fear extinction recall (Fig. 4d). Postmortem analysis of GRIN lens implantation site revealed positioning within the CeA of all six mice used for calcium signal and behavioral analysis (Supplementary Fig. 1a).

All miniscope implanted mice underwent habituation (5-CS only presentations) followed by acquisition training (5-CS/US pairings), extinction (30-CS only presentations), and extinction recall (4-CS only presentations, Supplementary Fig. 1b) on successive days. Similar to mice in Fig. 1, we observed no significant difference in behavioral responses during acquisition (Two-way repeated measures ANOVA, group × time interaction, F_*(4,16)*_ = 0.16, *P* = 0.96). We also did not observe significant differences during extinction training (Two-way repeated measures ANOVA, group × time interaction, F_*(9,36)*_ = 0.47, *P* = 0.89); however, we did observe a significant difference during extinction recall (Unpaired Student’s *t*-test, t_*(4)*_ = 2.87, *P* = 0.046). We observed a number of CeA–CRF neurons displayed an initial response to the CS presentation that rapidly habituated in both groups of mice (Fig. 4e). During acquisition training, we observed a number of neurons activated during the conditioning trials (Fig. 4f; 74 of 125, immediate extinction group; 84 of 119, delay extinction group). Within the population of responsive neurons, we observed three different response profiles, CS-only responsive (*n* = 6 cells, immediate extinction group and *n* = 13 cells delay extinction group), US-only responsive (*n* = 12 cells immediate extinction group and *n* = 7 cells delay extinction group), and neurons that initially responded to the US but transitioned to responding to the CS (*n* = 56 cells, immediate extinction group and *n* = 64 cells, delay extinction group; Supplementary Fig. 1c–e). Cells with distinct response profiles were broadly distributed throughout the field-of-view (FOV, Supplementary Fig. 1d). As a whole, cells that transitioned from US-responsive to CS-responsive during acquisition training showed a significant negative correlation between conditioning trial and US response amplitude and a significant positive correlation between conditioning trial and CS response amplitude (Supplementary Fig. 1f). Loss of US-responsiveness was similar to the emergence of CS-evoked freezing (Supplementary Fig. 1g); however, emergence of CS-responsiveness in these cells was delayed relative to freezing (Supplementary Fig. 1 g).

During extinction training, we observed equivalent CS-evoked calcium signals early in conditioning in mice undergoing immediate or delay extinction training (Fig. 4g). As conditioning progressed, we observed a significant difference in the slopes of the linear regression of the calcium signal and extinction trials between the delay extinction group and in the immediate extinction group (Fig. 4g, F_*(1,16)*_ = 8.29, *P* = 0.01). We also observed a significant difference in the average calcium signal in the delay extinction group compared to the immediate extinction group during extinction recall testing (Fig. 4h, unpaired Student’s *t*-test, t_*(92)*_ = 2.37, *P* = 0.02). To determine whether CS-evoked calcium signals could be used to classify mice as being in the immediate or delay extinction groups, we applied a Gaussian Naïve Bayes decoder^27^ to the signals from CS-responsive cells during habituation, acquisition, (Supplementary Fig. 2), extinction, and recall (Supplementary Fig. 3). Both extinction training and recall had significant decoding accuracies relative to the null distribution formed by randomly shuffling the identity of the recorded neurons^28^. No such significant decoding was observed during habituation or acquisition training (Fig. 4i).

**Fig. 5:**
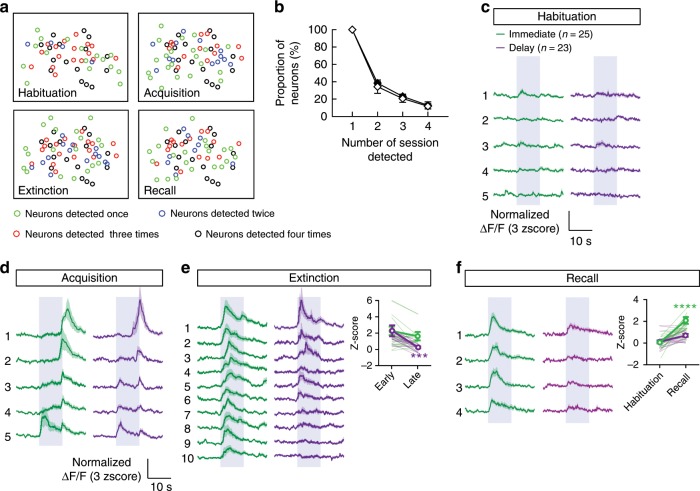
Longitudinal analysis of calcium signals in CeA–CRF neurons. **a** Maps of active neurons that were registered as same neurons across multiple imaging sessions. **b** Proportion of all active neurons detected across multiple sessions for two groups of mice that underwent immediate or delay extinction training (±S.E.M.). **c**–**f** Average calcium responses (±S.E.M.) of same cells during habituation (**c**), acquisition (**d**), extinction (**e**, average of 3-trial bins, inset: average Z-score responses early (first nine trials) and late (last nine trials); two-way repeated measures ANOVA, effect of time F_*(1,46)*_ = 13.09, ***P = 0.0007, delay early versus delay late *P* < 0.001), and recall (f, inset: average Z-score responses early (first nine trials) and late (last nine trials); two-way repeated measures ANOVA, effect of time F_*(1,46)*_ = 46.91, *P* < 0.0001, immediate habituation (hab.) versus recall, ****P < 0.0001).

To better resolve the plasticity of calcium signals in CeA–CRF neurons, we performed single-cell registration^29^ for the longitudinal analysis of the same cells across all sessions (Supplementary Fig. a–h). The proportion cells that could be registered as the same and detected as active across all sessions declined dramatically (Fig. 5a, b), consistent with previous longitudinal imaging of amygdala neurons of the BLA^30^. Longitudinal analysis of CeA–CRF neurons revealed that cells that acquired CS-responsiveness during acquisition training displayed little response to the CS during habituation and initially responded to the US early in conditioning (Fig. 5c, d). CS-responsive neurons in the immediate extinction group maintained equivalent responding from early to late extinction training and remained above habituation levels of responding during recall (Fig. 5e). In contrast, CS-responsive neurons in the delay extinction group displayed significantly reduced calcium signals from early compared to late extinction and the level of responding during recall was equivalent to habituation levels (Fig. 5f).

### Inhibition of CS activity attenuates extinction deficits

**Fig. 6:**
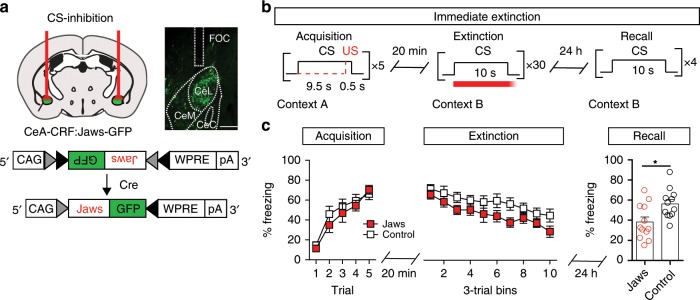
Inhibition of CS-evoked activity attenuates the immediate extinction deficit. **a** Schematic of bilateral AAV1-FLEX-Jaws-GFP and optical fiber placement with representative histological verification of Jaws-GFP expression and cannula placement (scale bar: 250 μm). Control mice were injected with AAV1-FLEX-GFP. **b** Schematic of fear conditioning paradigm showing Jaws inhibition during CS presentation (*n* = 12/group). **c** Freezing responses to CS during acquisition (left), immediate extinction (middle), and extinction recall (right, Unpaired Student’s *t*-test, *P < 0.05). Data are presented as mean ± S.E.M.

The persistence of CS-evoked increases in calcium signals during immediate extinction and extinction recall suggests that this activation is critical for the observed necessity of CeA–CRF neurons in driving the immediate extinction deficit. To test this more directly, we conditionally expressed the inhibitory opsin Jaws^31^ in the CeA–CRF neurons by injecting CRF-Cre mice bilaterally into the CeA with AAV1-FLEX-Jaws-GFP (Fig. 6a, *n* = 12 mice) or AAV1-FLEX-GFP (control, *n* = 12 mice) and implanting bilateral optical cannulas (Fig. 6a and Supplementary Fig. 5a). Mice underwent fear conditioning as above, followed 20 min later by fear extinction training. During extinction training, red light (640 nm) was delivered to the CeA at CS-onset for the duration of the CS presentation (10 s) followed by a 1 s ramp-down to prevent rebound excitation associated with delivery of a square light pulse^32^. Twenty-four hours following extinction training mice were tested for extinction recall (Fig. 6b). Acquisition of CS-evoked freezing did not differ between groups (Fig. 6c, two-way repeated measures ANOVA, group × time interaction, F_*(4,88)*_ = 0.47, *P* = 0.76). Freezing during extinction training also did not differ between CeA–CRF:Jaws-GFP and CeA–CRF:GFP mice (Fig. 6c, two-way repeated measures ANOVA, group × time interaction, F_*(9,198)*_ = 0.43, *P* = 0.92). During extinction recall we observed a significantly less CS-evoked freezing in CeA–CRF:Jaws-GFP compared to CeA–CRF:GFP mice (Fig. 6c, unpaired Student’s *t*-test, t_*(22)*_ = 2.75, *P* = 0.012).

### CeA–CRF neurons are sufficient to drive fear reinstatement

Following extinction of a CR to a CS, the CR can be reinstated by presentation of an unpaired US^33^. Antagonism of centrally acting CRF is also sufficient to prevent US-induced reinstatement^34^. We observed a small number of CeA–CRF neurons that responded to the US throughout conditioning (12 of 125 immediate extinction group and 7 of 119 delay extinction group), and a larger number of CeA–CRF neurons initially responded to the US and transitioned to the CS (56 of 125 immediate extinction group and 64 of 119 delay extinction group). The US-responsiveness of these neurons and there persistent activation to the CS in immediate extinction suggest that they may be sufficient to reinstate an extinguished fear memory.

**Fig. 7:**
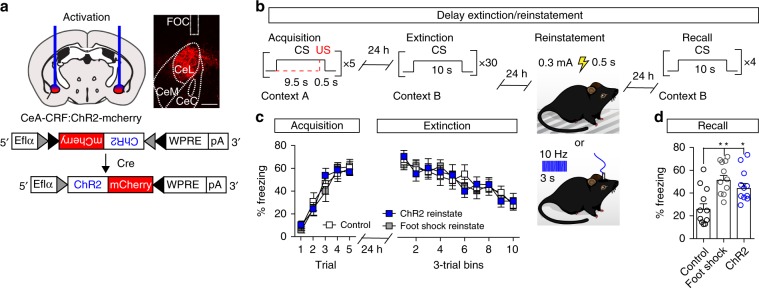
CeA–CRF neurons mediate fear reinstatement. **a** Schematic of AAV1-FLEX-ChR2-mCherry expression and bilateral optical fiber placement in the CeA of CRF-Cre mice with representative histological verification of ChR2-mCherry expression and cannula placement. Control mice and US reinstatement mice were injected with AAV1-FLEX-mCherry. **b** Schematic of fear conditioning paradigm. Following delay extinction mice were given a single US presentation (0.3 mA, 0.5 s) or a single optical stimulation (10 Hz, 3 s) in the extinction context (*n* = 11/group). **c** Freezing responses to CS during acquisition (left) and delay extinction (right). **d** Freezing to CS presentation during extinction recall following reinstatement (One-way ANOVA followed by Bonferroni multiple comparisons, **P < 0.01, *P < 0.05). Data are presented as mean ± S.E.M.

To test whether activation of CeA–CRF neurons following extinction training is sufficient to reinstate a CR to the CS, we conditionally expressed ChR2^35^ in these cells by bilateral injection of AAV-FLEX-ChR2-mCherry or AAV1-FLEX-mCherry (control) into the CeA of CRF-Cre mice (Fig. 7a) and placed optical cannulas bilateral over the CeA (Fig. 7a and Supplementary Fig. 5b). Mice underwent fear conditioning with 5-CS/US parings (0.5 mA, 0.5 s foot shock) followed 24 h later by delay extinction training. Twenty-four hours after extinction training, mice received a single US presentation (0.3 mA, 0.5 s foot shock) with optical stimulation of mCherry (US reinstatement group; *n* = 11 mice), a single stimulation of ChR2 in CeA–CRF neurons (473 nm, 5 ms pulse-width, 10 Hz for 3 s; CeA–CRF reinstatement group; *n* = 11 mice), or a single stimulation of mCherry (no reinstatement control group; *n* = 11 mice) (Fig. 7b). There was no difference between groups during acquisition (Fig. 7c, two-way repeated measures ANOVA, group × time interaction, F_*(8,120)*_ = 0.51,*P* = 0.85), or extinction training (Fig. 6c, two-way repeated measures ANOVA, group × time interaction, F_*(18,270)*_ = 0.98,*P* = 0.48). Following reinstatement, we observed significantly higher CS-evoked freezing in the foot shock and CeA–CRF stimulation groups compared to control mice (Fig. 7d, one-way ANOVA, F_*(2,30)*_ = , *P* = 0.0012).

## Discussion

Our results demonstrate that persistent activation of CeA–CRF neurons during immediate extinction training and during extinction memory recall is associated with an increased freezing during in the extinction memory recall relative to mice that undergo delay extinction training. Impairments in extinction memory recall are dependent on the activity of CeA–CRF neurons and the production of CRF. Activation of CeA–CRF cells following extinction training is sufficient to reinstate a previously extinguished fear response. These results are consistent with CeA–CRF neurons being an important contributor to the stress-arousal systems associated with the recency of fear that has been proposed to underlie the immediate extinction deficit^6^.

We observed a relatively large proportion of CeA–CRF neurons (49% of the total population recorded) transitioned from responding to the US to responding to the CS during conditioning (Supplementary Fig. 1c–g). In contrast, PKCδ-expressing neurons of the CeA were shown to largely respond to the US (~49%) and displayed little transition to the CS during conditioning, though these cells did respond to the CS during recall that attenuated during the course of a short extinction training^23^. Collectively, these data support previous observations that CeA–CRF neurons and CeA-PKC PKCδ neurons are largely distinct^15,36^. Although calcium responses have been observed in CeA-SOM in response to an aversive CS^22^, how these neurons respond during acquisition and extinction is not yet resolved; thus, we cannot make additional conclusions about the relatedness of CeA–CRF and CeA-SOM neurons in this context.

Consistent with previous reports, we did not observe significant differences in the decrement of CS-evoked freezing during extinction training between immediate and delays extinction groups^3–5,7,8^. In several cases, disruption of CeA–CRF neuron activity appeared to display a trend towards reduced freezing during extinction training (Figs. 1–3, and 6); however, analysis of all groups that had a disruption of CeA–CRF neuron activity during immediate extinction training (*n* = 44 total) revealed equivalent reductions in freezing during extinction training relative to controls (*n* = 44 total, two-way repeated measures ANOVA, group × time interaction, F_*(9,774)*_ = 0.42, *P* = 0.93).

It has been proposed that impairments in extinction memory recall may result from either impairment in extinction memory consolidation or a generalization effect that impairs extinction retrieval^7^. Our experiments do not address this issue directly; however, our previous analysis of CeA–CRF neurons demonstrated that these cells do not influence conditioned threat generalization^15^ suggesting that these neurons either antagonize the consolidation of inhibitory extinction memories or attenuate the weakening of the fear acquisition memory. CS-evoked freezing during extinction training was not affected by the recency of fear conditioning; however, CS-evoked calcium signals in CeA–CRF neurons showed a significant interaction between the fluorescent signal in the immediate and delay extinction groups and extinction training (Supplemental Fig. 1h; two-way ANOVA, F_*(9,1430)*_ = 2.31, *P* = 0.014). We also observed that CS-evoked calcium signals in CeA–CRF neurons emerged delayed relative to CS-evoked freezing. Collectively these data support previous observations that CeA–CRF neurons do not drive freezing behavior per se, but rather provide an important modulatory signal for the regulation of fear-associated behavior^14,15^.

Consistent with CRF being and important modulatory system for regulating fear, CRF signaling enhances synaptic strength in the CeA and serves as a gain control for the formation of fear memories when threat intensities are low^15^. If the expression of fear extinction is the result of a combined weakening of the fear memory trace and the formation of a competing inhibitory extinction memory trace^2^, then maintenance of a signal that promotes synaptic strengthening associated with the fear memory would antagonize the stable weakening of the fear memory trace and prevent the extinction memory from being fully expressed. Our observation that CS-evoked freezing can be reinstated by a single activation of CeA–CRF neurons 24 h prior to extinction retrieval testing, suggests that the signaling of CRF may rapidly re-strengthen the previously weakened fear memory trace. Previous observations that delivery of a US just prior to extinction retrieval following delay extinction training impairs the extinction memory recall^7^ further supports effects of immediate extinction training on the fear memory itself rather than an impairment in consolidation of the extinction memory. Given that CeA–CRF has been linked to stress-induced reinstatement of drug-seeking behavior^37^, similar mechanisms of CRF are likely at play. Future experiments investigating the role of CRF in these processes will shed additional light on this topic.

It has been shown that persistent CS-evoked activity in the infralimbic cortex is associated with immediate extinction deficits^3^. Systemic antagonism of noradrenergic signaling reduces fear-induced activity in the prelimbic and infralimbic cortex and rescues immediate fear extinction deficits^4^. Interestingly, direct antagonism of noradrenergic signaling in the basolateral amygdala (BLA), but not the prefrontal cortex, rescues the immediate extinction deficit^5^. CeA–CRF neurons potently enhance the activity of neurons in the LC, a major site of noradrenaline production in the brain^16^. LC noradrenergic neurons have opposing effects on fear memory acquisition and extinction that is dependent on projections to either the BLA or infralimbic cortex^38^. Similar to CeA–CRF neurons, BLA projecting LC noradrenergic neurons influence the acquisition of the fear memory. However, temporally precise inhibition of LC noradrenergic projections to the BLA during delay extinction training enhanced fear extinction memory recall without affecting extinction training^38^. We also observed that TeTx silencing of CeA–CRF neurons enhance extinction recall following delay extinction training (Fig. 1c). Given that we observed some residual activation of CeA–CRF neurons during fear recall following delay extinction training (Fig. 3h), this likely reflects some persistence of fear even after a 24 h delay. Thus, reducing this residual activation further with TeTx inactivation further enhanced fear extinction memory recall. Based on this collective data, it is likely that CeA–CRF neurons projecting to the LC enhance the activity of noradrenergic LC neurons projecting to the BLA that provides a tripartite stress/arousal circuit that promotes the persistence of fear. In summary, we propose that CeA–CRF neurons reflect this persistent state of fear and through the modulatory actions of CRF signaling prevents the weakening of the fear memory that directly antagonizes the recall of the fear extinction memory.

## Methods

### Animals

Male and female *Crh*^*IRES-Cre*^ mice were group-housed on a 12 h light/dark cycle with free access to food and water. Mice were randomly assigned into treatment groups and behavioral experiments were conducted during the light cycle (lights on at 7 am). All procedures were approved by the University of Washington Animal Care and Use Committee. Equal numbers of male and female mice were used for all experiments.

### Virus production and surgery

AAV1-FLEX-GCaMP6m, AAV1-FLEX-GFP-TeTx, AAV1-FLEX-hM4Di-YFP, AAV1-FLEX-hM3Dq-mCherry, AAV1-FLEX-Jaws-GFP, AAV1-FLEX-ChR2-mCherry, AAV1-FLEX-YFP, and AAV1-FLEX-mCherry were produced in house (titer ~3 × 10^12^ particles/µl) as described^39^. Briefly, AAV shuttle plasmids are co-transfected into HEK293T/17 cells (ATCC CRL-11268) with the AAV packaging plasmid pDG1. Forty-eight hours post-transfection cells were harvested and virus was liberated by three-repeated freez-thaw cycles. Vectors were purified by sucrose and cesium gradient centrifugation, dialyzed against Hank’s balanced salt solution, pelleted by sucrose gradient centrifugation and resuspended in Hank’s balanced salt solution. Viral vectors were stored at −80 °C before surgery. Viral vectors were stereotaxically injected (0.5 μl) at a rate of 0.25 μl/min bilaterally into the CeA of isofluorane-anesthetized mice (2–4 month old) using the following coordinates: −1.2 mm posterior, ±2.9 mm lateral, and −4.5 mm ventral to bregma. For Jaws- or ChR2-injected mice, bilateral optic fibers were implanted dorsal to the CeA (−4.0 mm). After the surgical procedures, all mice were allowed to recover for 2–3 weeks. For calcium imaging, GCaMP6m-injected mice were anaesthetized again and implanted with a microendoscope lens (6.1 mm length, 0.5 mm diameter; Inscopix, #100-000588) while fluorescent signals from CRF neurons were visualized using nVista acquisition software (Inscopix). GCaMP6m-expressing mice were co-injected with AAV1-FLEX-hM3Dq-mCherry and calcium signals were elicited during GRIN implantation by injection of CNO (1 mg/kg). One week following GRIN implantation, a baseplate (Inscopix, #100-000279) was mounted at a location above the lens that provided the best field of view.

### Fear conditioning

Acquisition of fear memory took place in four identical chambers (21.6 × 17.8 × 12.7 cm; Med Associates Inc.) located in sound-attenuating boxes. Each chamber was equipped with a house light, a speaker, and a metal-grid floor (context A). The chamber was cleaned with a 1% acetic acid solution between animals and a container filled with the solution was placed under the floor. Fear responses to CS were tested in a perceptually different environment in which white plastic panels were inserted to cover the walls and floor (context B). The panels were also wiped with 70% ethanol between mice.

Fear conditioning procedures began with measuring basal freezing responses to CS (10 kHz; 10 s) in context B. Five CS trials were given with an average inter-trial interval of 80 s ranging 60–100 s. On the next day, mice received five CS trials which co-terminated with a 0.5 s foot shock (0.5 mA) in context A. After conditioning, the mice were returned to their home cages. Either 20–30 min or 24 h later, the mice underwent 30 CS-alone trials in context B for extinction training, extinction trials were averaged across three-trial bins for statistical analysis. For hM3Dq- and hM4Di-injected mice, CNO (1 mg/kg) was intraperitoneally administered 30 min prior to extinction training. On the following day of extinction, the mice were exposed to four CS presentations to test the recall of extinction memory. For optical inhibition and stimulation, only optic fibers with >70% power retention were used. For inhibition, a red light pulse (640 nM laser, Laserglow Technologies at 10 mW power) was delivered 0.1 s prior to CS onset for a period of 10 s followed by a 1 s ramp-down. For excitation, blue light stimulation (473 nm laser, Laserglow Technologies at 10 mW power) for 3 s was delivered at 10 Hz, using a 5 ms pulse-width. During the experiments, each mouse’s behavior was video-taped using a camera mounted above the chamber. Movement velocities were measured offline using a video tracking software (EthoVision XT 8.5, Noldus Technology). Freezing behavior in response to CS was scored if movement velocities were less than 0.75 cm/s for at least 1 s.

### Calcium imaging

Calcium signals from GCaMP6m-injected mice were acquired at 10 Hz using nVista acquisition software (Inscopix) while they underwent the same conditioning procedures except for an average inter-trial interval of 90 s. The LED (40–60% power) of the miniature microscope was turned on by a TTL pulse from the Med Associates chamber 30 s before CS onset and off after 20 s after the onset. Imaging data for each 50 s epoch were downsampled (spatial binning factor: 4; temporal binning factor: 2) and sequentially concatenated. Concatenated, downsampled video files where then motion corrected using Mosaic software (Inscopix). Then calcium transients (∆F/F) of individual CRF neurons were extracted with a constrained non-negative matrix factorization algorithm for microendoscopic data (CNMF-E)^40^. Their spatial locations were further analyzed with a cell registration method^29^ to identify the same neurons detected across all four imaging sessions. The method uses a probabilistic approach to register as same or different cells based on the distributions of centroid distances and spatial correlations between all neighboring cell-pairs from different imaging sessions.

To examine CRF neuronal activity during fear conditioning, CS (0–5 s epoch from CS onset) and US (9.5–13 s epoch from CS onset) responses were averaged within a given imaging session and compared to baseline activity in the last 10 s of the pre-CS period using Wilcoxon signed-rank tests. CRF neurons with significantly higher activity during each epoch were categorized as responsive. Neuronal calcium transients were transformed to z-scores for the designated periods (0–5 s epoch from CS onset and 9.5–13 s epoch from CS onset for the US response) relative to the basal pre-CS activity.

### Decoding analysis

The fluorescence values per frame for 7 s of pre-cue baseline and 9 s of cue (0.5 s prior to US presentation) activity for each trial were counted as a response feature of a neuron. To include all trials, we concatenated the fluorescence for the above 16 s window for every trial. The dimensionality of this response feature space was then reduced using principal component analysis (using Scikitlearn in Python) applied to all recorded neurons per learning stage (i.e., pooled between the immediate and delay extinction groups). The number of principal components retained was decided by identifying a bend in the plot of the variance explained per principal component—the scree plot^26^. The response features of each neuron were then projected onto this principal component space to obtain the reduced dimensionality feature space. A Naïve Bayes Gaussian classifier (GaussianNB in Scikitlearn) was then applied to this dataset to test for decoding. The accuracy of decoding was calculated as mean accuracy per test split using 5-fold cross-validation, using the cross_val_score function in Scikitlearn. To test whether the observed cross-validated accuracies could result from chance, we compared this accuracy to the null distribution of accuracies obtained while shuffling the identities of neurons between the immediate and delayed extinction groups. A total of 4000 shuffles were performed per condition. The percentile of the true accuracy relative to this null distribution was calculated as the one-tailed p-value (a priori hypothesis that prediction accuracy is higher than null). Since a total of four comparisons were made (habituation, acquisition, extinction and recall), Benjamini-Hochberg correction was used to correct p-values for these multiple comparisons.

### Immunohistochemistry

Mice were transcardially perfused with phosphate-buffered saline (PBS) and 4% paraformaldehyde (PFA). Their brains were extracted, post-fixed in the PFA at 4 °C overnight, and cryoprotected in PBS containing 30% sucrose for 72 h. Then the brains were frozen and cut in coronal sections (30 µm) on a cryostat (Leica CM 1850). The sections were treated in 0.3% Triton-X PBS with 3% normal donkey serum for 1 h and incubated at 4 °C overnight with the following primary antibodies: (1) anti-GFP (polyclonal, 1:2000; Invitrogen, A11122) for GCaMP6m-, HM4-, and Jaws-injected mice, (2) anti-dsRed (polyclonal, 1:1000; Clontech, 632496) for HM3- and ChR2-injected mice. After rinsing with PBS three times for 10 min each, the sections were incubated with secondary antibodies conjugated to AlexaFluor 488 or CY3 (donkey anti-rabbit, 1:200; Jackson immunoresearch) for 1 h at room temperature. The sections were then washed in PBS three times for 10 min, mounted on glass slides, and coverslipped with Fluoromount-G (Southern Biotech). Using a Nikon upright microscope, fluorescent images were taken and examine protein expression levels, imaging sites, and fiber placements.

### Statistical analysis

Calcium imaging and behavioral results were analyzed with mixed-design ANOVA that contained within-subjects variables (e.g., trial) and between-subjects factors (e.g., group). Group comparisons were also performed with one-way ANOVA. When significant interactions were detected, Bonferroni corrected *t*-tests were used for post-hoc pairwise comparisons. Pearson’s Spearman’s correlation tests were conducted to establish a relationship between two variables. Two-tailed *P*-values < 0.05 were considered statistically significant. Data were expressed as mean ± SEM.

## Supplementary information

Supplementary Information

## Data Availability

Unrestricted access to all minimal data required for the interpretation or replication of the findings, or to build upon the methods reported in this article will be made available upon request to the corresponding author.

## Code availability

Analysis codes for calcium imaging can be found at the following GitHub links: (1) CNMF-E, https://github.com/zhoupc/CNMF_E; (2) cell registration, https://github.com/zivlab/CellReg.
