# Supplementary material for: Persistent activation of central amygdala CRF neurons helps drive the immediate fear extinction deficit
Source: Nat Commun. 2020 Jan 22;11:422. doi: 10.1038/s41467-020-14393-y (PMC6976644; doi:10.1038/s41467-020-14393-y)
Supplement: Supplementary file 1 — Supplementary Information [file 41467_2020_14393_MOESM1_ESM.pdf]

Persistent activation of central amygdala CRF neurons helps drive the immediate fear extinction deficit

Yong S. Jo, Vijay M.K. Namboodiri, Garret D. Stuber, and Larry S. Zweifel

Supplementary information

Supplementary figure 1-5

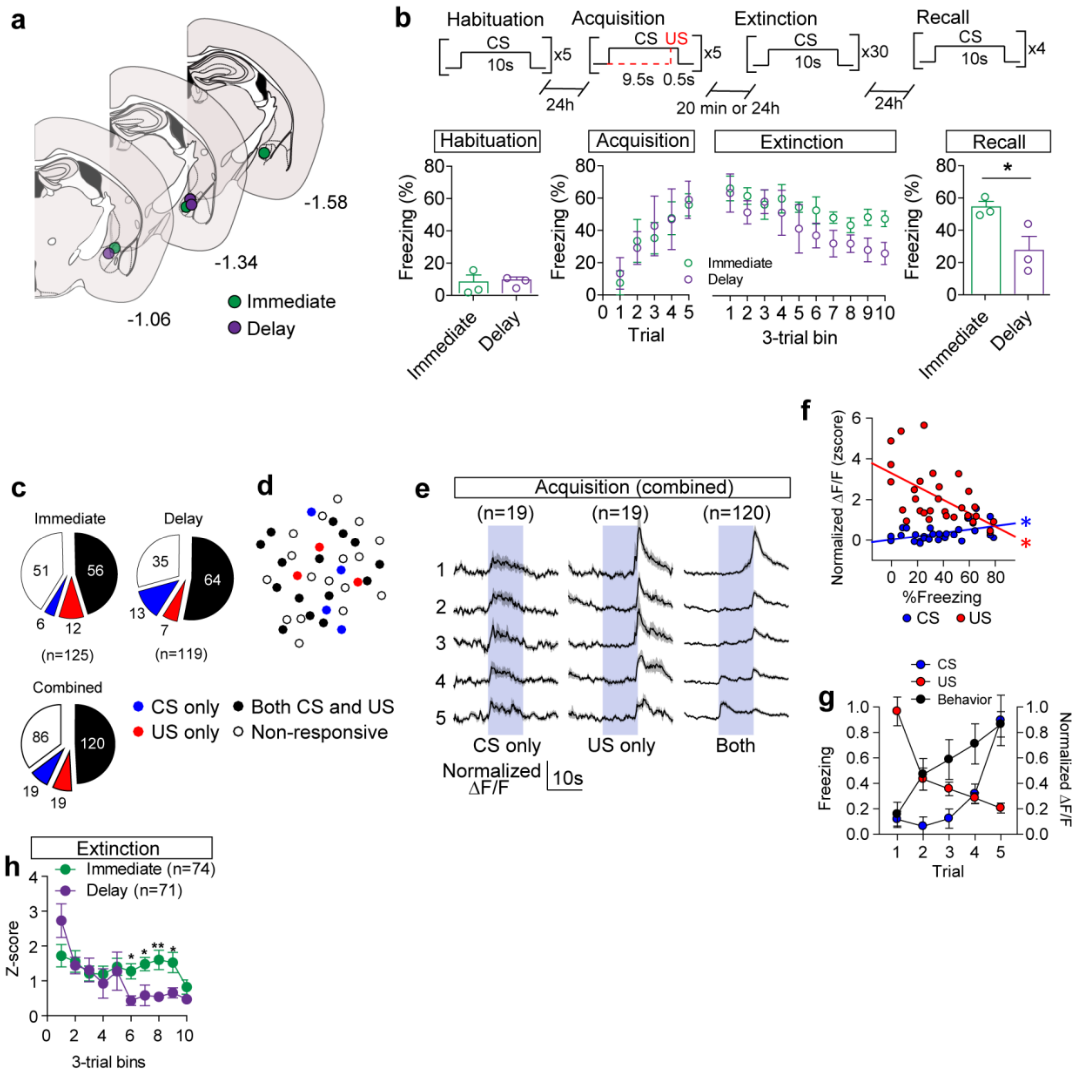

Supplementary Figure 1 Extended data for *in vivo* imaging of CeA-CRF neurons. (a) Anatomical placement of GRIN lenses in mice from immediate and delay fear extinction groups (n=3/group). Values are distance (mm) posterior to Bregma. (b) Behavioral paradigm used for imaging CeA-CRF neurons during extinction (top). Habituation, acquisition, and extinction did not differ in mice imaged during either immediate or delay extinction training, but recall was significantly different between the groups (bottom, unpaired students t-test, *P<0.05). (c) Proportion of neurons that responded to the CS only, US only, both CS and US or were non-responsive during acquisition training in the immediate and delay extinction groups. (d) Spatial location of

responsive and non-responsive cells during acquisition training from an example mouse. (e) Composite acquisition data from the two groups of mice showing the average responses of CS-only, US-only, and both CS and US-responsive cells. (f) Composite of normalized calcium signals to the US and CS in cells responding to both the CS and US during acquisition versus freezing behavior (Pearson r, *P<0.05). (g) Composite of normalized calcium signals to the US and CS in cells responding to both the CS and US during the five conditioning trials relative to freezing in those trials. (h) Average z-score responses of activated cells during extinction training in immediate and delay extinction trained mice (two-way ANOVA, $F_{(9,1430)}$=2.31, P=0.014; followed by Bonferroni multiple comparisons, **P<0.01, *P<0.05). Data are presented as mean±S.E.M.

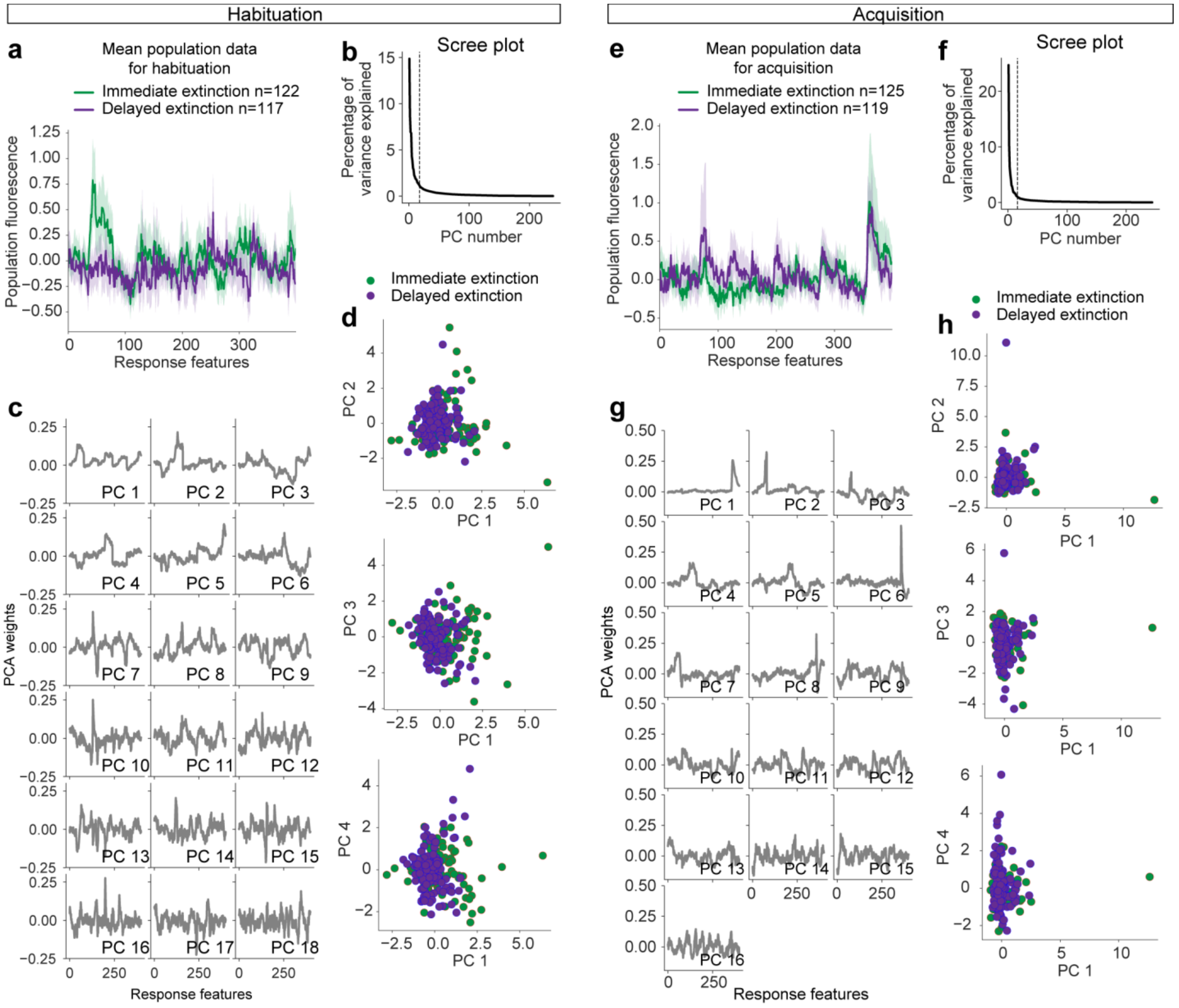

Supplementary Figure 2 Extended data for Gaussian decoder analysis during habituation and acquisition in immediate and delay fear extinction groups. (a) For each neuron, 7 s of pre-cue baseline and 9 s of cue presentation for each trial (recorded at 5 Hz) were included as a "response feature". During habituation, all 5 trials were concatenated together to provide the complete set of "response features" (x-axis) per neuron. The average and 95% confidence intervals (in shading) of these response features across all neurons in the immediate or delayed extinction groups are shown in red and blue respectively. The legend shows the number of neurons per group. (b) The dimensionality of this response feature space was then reduced using principal component analysis (Methods). The variance explained for each principal component is shown as a scree plot, with the number of principal components retained shown by the

dashed line. Each retained principal component vector is shown in (c). (d) A scatter plot of the principal component projections for all recorded neurons is shown across a space formed by the first four principal components. (e-h) Same as in (a-d), but for recordings during acquisition training.

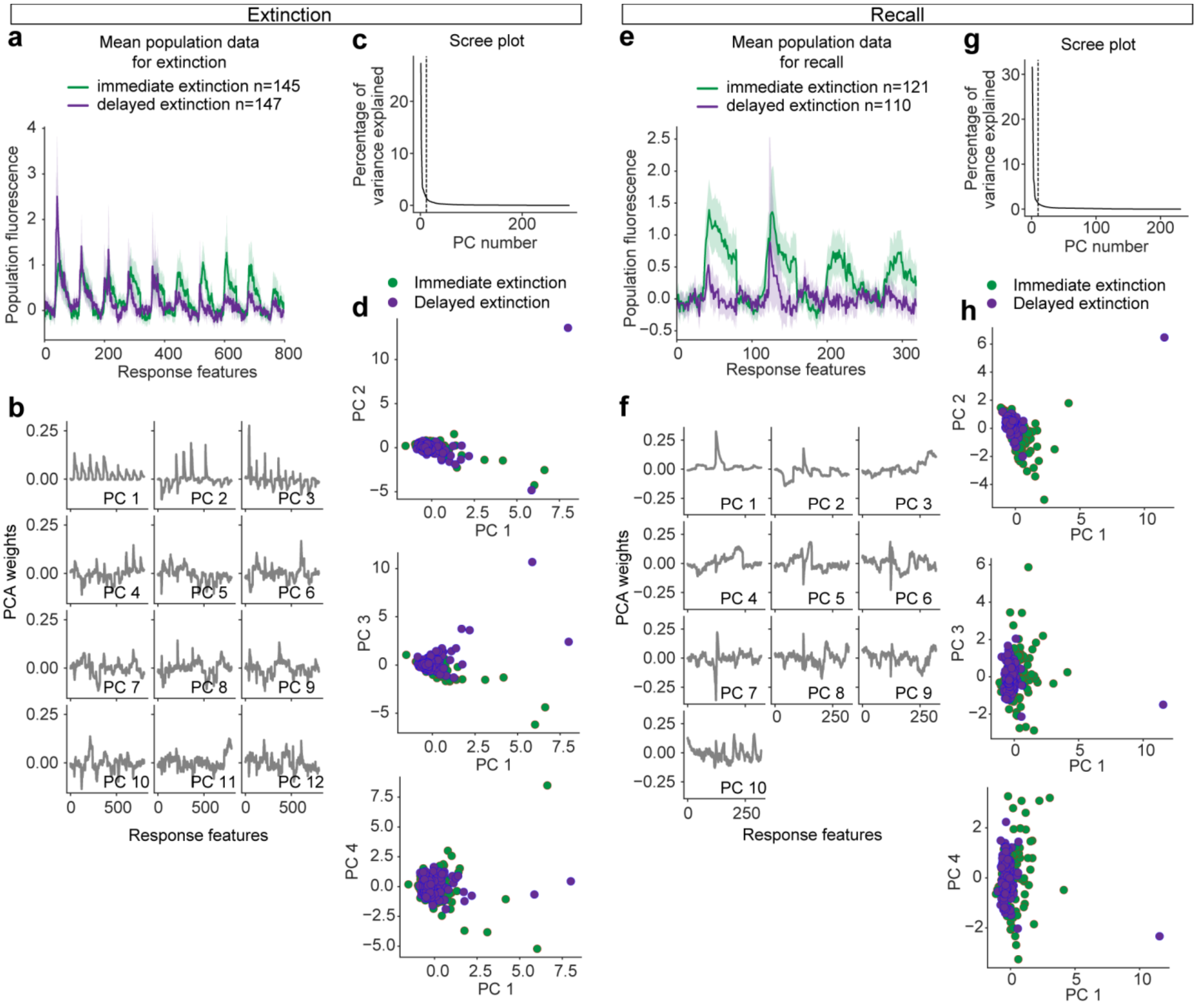

Supplementary Figure 3 Extended data for Gaussian decoder analysis during extinction and recall in immediate and delay fear extinction groups. Same as in Supplementary Fig 2, but for recordings during extinction and recall.

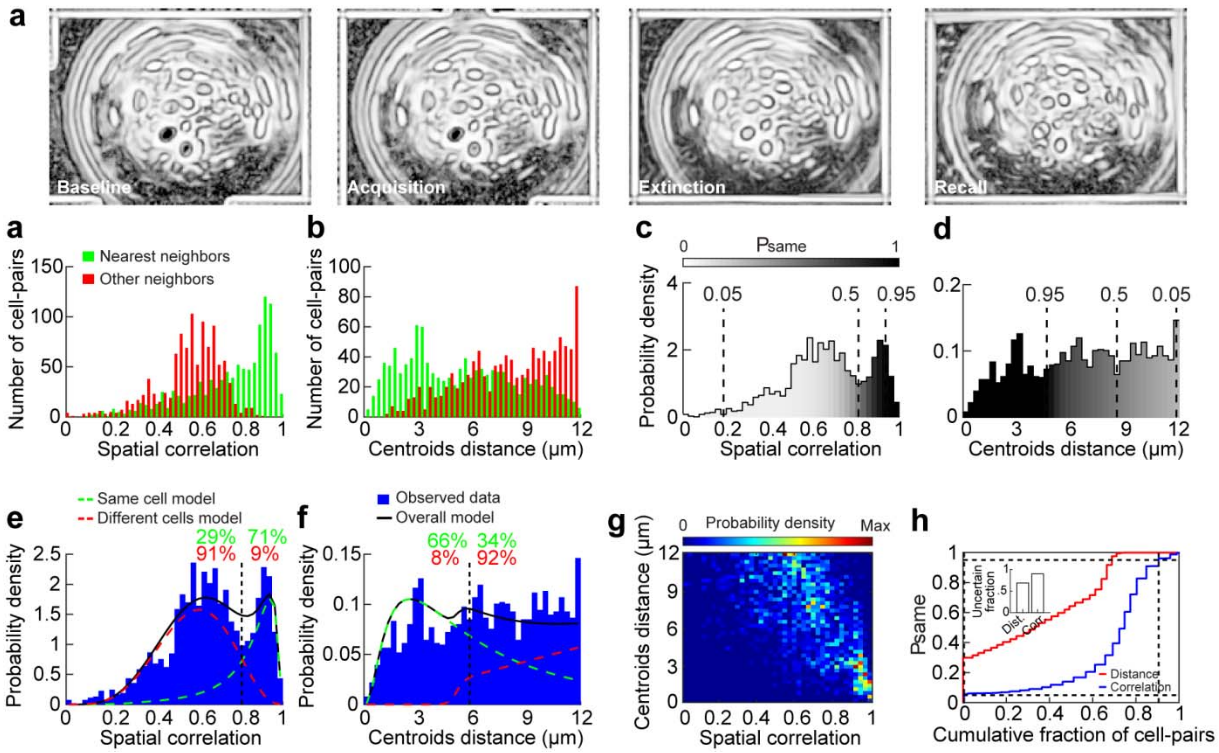

Supplementary Figure 4. Single cell registration analysis. (a) Locations of GCaMP6m-expressing neurons recorded across four behavioral sessions from a representative mouse. Neuron outlines were visualized with the CNMF-E algorithm by calculating temporal cross-correlation of each pixel with its adjacent pixels. The location information was further processed with the cell registration method to identify the same neurons recorded during all behavioral sessions. (b) Distributions of spatial correlations between cell-pairs of nearest neighbors (green, same cell candidates) and other neighbors (red, different cell candidates) across the four sessions. (c) and centroid distances between cell-pairs of nearest neighbors (green, same cell candidates) and other neighbors (red, different cell candidates) across the four sessions. (d) Distributions of spatial correlations between all neighboring cell-pairs (blue bars) and the modeled distributions of same cells (dashed green line), different cells (dashed red line), and their weighted sum (solid black line). (e) Same as in (d) for centroid distances. (d-e) Vertical black dashed lines indicate the intersection between the two models. (f-g) Distributions of spatial correlations (f) and centroid distances (g) between neighboring cell-pairs. The grayscale color displays the probability for two cells from two imaging

sessions to be the same cell (P_{same}). (h) Color-coded joint distribution of measurements for all cell-pairs (spatial correlations and centroid distances). (i) Cumulative fraction of same cells estimated by spatial correlations and centroid distances. The cumulative fraction of uncertain cell-pair registrations ($0.05 \leq P_{same} \leq 0.95$) was smaller in the centroid distances model; thus, the cell-pairs that had centroid distances shorter than 6 μm (the intersection point in (e)) were considered the same cell in two imaging sessions.

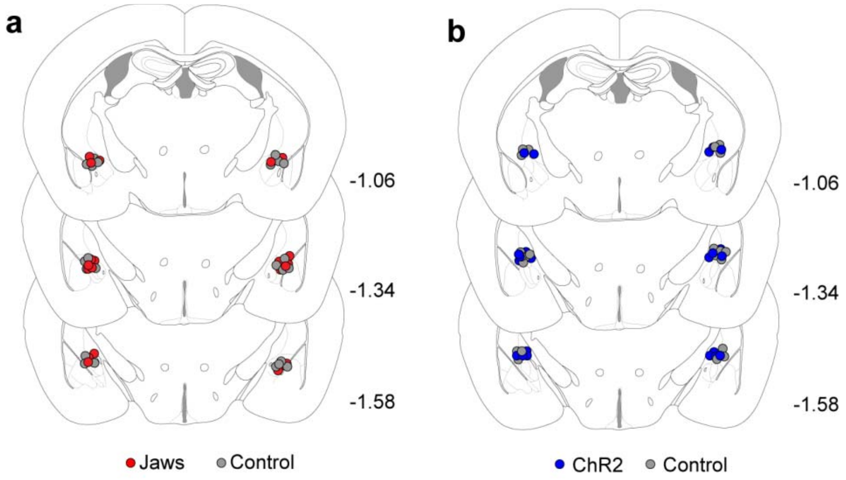

Supplementary figure 5. Optical fiber placement in Jaws-YFP and ChR2-mCherry expressing mice. (a) Optical fiber placement in CeA of CRF-Cre mice injected with AAV1-FLEX-Jaws-YFP or YFP control (n=12/group). (b) Immunohistochemistry showing Jaws-YFP expression in the CeA and optical fiber placement. Scale: 250 μm. (c) Optical fiber placement in CeA of CRF-Cre mice injected with AAV1-FLEX-ChR2-mCherry or mCherry control (n=11/group). (d) Immunohistochemistry showing ChR2-mCherry expression in the CeA and optical fiber placement. Scale: 250 μm.
